# Calcyclin-binding protein contributes to cholangiocarcinoma progression by inhibiting ubiquitination of MCM2

**DOI:** 10.32604/or.2023.028418

**Published:** 2023-05-24

**Authors:** YUSEN ZHANG, LIPING LIU, BIWEI LUO, HONGGUI TANG, XIAOFANG YU, SHIYUN BAO

**Affiliations:** 1 Department of Hepatobiliary and Pancrease Surgery, The Second Clinical Medical College, Jinan University (Shenzhen People’s Hospital), Shenzhen, 518020, China; 2 Department of General Surgery, The Second Clinical Medical College, Jinan University (Shenzhen People’s Hospital), Shenzhen, 518020, China; 3 Department of Hepatobiliary and Pancrease Surgery, The First Affiliated Hospital, Southern University of Science and Technology, Shenzhen, 518020, China

**Keywords:** CACYBP, CCA, Ubiquitination, Wnt/β-catenin pathway, Prognosis

## Abstract

**Background:**

Cholangiocarcinoma (CCA) represents the epithelial cell cancer with high aggressiveness whose five-year survival rate is poor with standard treatment. Calcyclin-binding protein (CACYBP) shows aberrant expression within several malignant tumors, but the role of CACYBP in CCA remains unknown.

**Methods:**

Immunohistochemical (IHC) analysis was used to identify CACYBP overexpression in clinical samples of CCA patients. Moreover, its correlation with clinical outcome was revealed. Furthermore, CACYBP’s effect on CCA cell growth and invasion was investigated *in vitro* and *in vivo* using loss-of-function experiments.

**Results:**

CACYBP showed up-regulation in CCA, which predicts the dismal prognostic outcome. CACYBP had an important effect on in-vitro and in-vivo cancer cell proliferation and migration. Additionally, knockdown of CACYBP weakened protein stability by promoting ubiquitination of MCM2. Accordingly, MCM2 up-regulation partly reversed CACYBP deficiency’s inhibition against cancer cell viability and invasion. Thus, MCM2 might drive CCA development by Wnt/β-catenin pathway.

**Conclusions:**

CACYBP exerted a tumor-promoting role in CCA by suppressing ubiquitination of MCM2 and activating Wnt/β-catenin pathway, hence revealing that it may be the possible therapeutic target for CCA treatment.

## Introduction

Cholangiocarcinoma (CCA) represents the epithelial cell malignancy that shows typical feature like cholangiocyte differentiation. According to its anatomic position, CCAs is divided into intrahepatic (iCCA), perihilar (pCCA), or distal CCA (dCCA) [1]. pCCA occupies the greatest proportion (50%) in CCA patients, whereas iCCA and dCCA represent 10% and 40% of CAA cases, respectively [2]. Epidemiological studies have shown that the overall CCA morbidity shows a growing trend during the last thirty years, and its five-year survival after diagnosis is 10% [3,4]. CCAs are an aggressive cancer hardly diagnosed in the earliest stages of the disease, and patients are often presented with an advanced-stage disease [5]. Surgical treatment has been the common treatment for CCA cases, although it is only appropriate for a few early CCA cases. The median overall survival (OS) for advanced-stage CCA cases is below one year, even though standard-of-care chemotherapy regimen is administered [6].

The survival outcome of patients affected by CCA might improve greatly by targeted therapy. For instance, NVP-BGJ398, a pan-FGER inhibitor, showed a disease-control rate of 82% [7]. However, long-term use of these small-molecule targeted compounds may lead to drug resistance in CCA patients, which greatly reduces treatment efficacy [8]. Therefore, uncovering more promising therapeutic targets to develop effective CCA treatments is paramount to improve survival rate in CCA patients.

Calcyclin-binding protein (CACYBP), referred to SIP as well, stands for the multi-ligand protein related to various cell activities, such as cell proliferation, differentiation as well as protein ubiquitination [9,10]. CACYBP includes three major domains: N-terminus, the central CS, together with C-terminus [11]. Through these domains, CACYBP can interact with E3 ligase-associated complexes, cytoskeletal proteins and S100 family proteins [10]. Numerous studies have highlighted the aberrant expression of CACYBP in several malignant cancers like glioma [12], breast cancer (BC) [13], gastric cancer (GC) [14], colon cancer [15] and pancreatic cancer [16]. Although CACYBP overexpression is associated with the development of these types of cancers, its effect on CCAs is still unknown.

## Materials and Methods

### Tissue microarray and immunohistochemistry (IHC)

Human microarray chip (Cat No. HBiDC122Su01) including CCA tissue as well as normal non-carcinoma tissue was provided by Shanghai Outdo Biotech Company. Corresponding information of patients and written informed consent were collected. This work conducted IHC analysis for detecting CACYBP levels within tumor and normal tissues. Briefly, xylene was added for tissue section dewaxing, while ethanol at different concentrations for rehydration. The antigen was repaired by citrate buffer following the blocking of 3% hydrogen peroxide solution. Subsequently, sections were then incubated using primary antibody CACYBP (1:50; Cat. #ab171972; Abcam, Cambridge, UK) together with horseradish peroxidase (HRP)-labeled goat anti-rabbit IgG secondary antibody (1:400; Cat. #ab97080; Abcam), followed by staining with diaminobenzene (DAB) as well as hematoxylin, observation in a microscope and evaluation independently by Haonon et al.’s method [37] via two pathologists.

### Bioinformatics analysis

Differential expression of CACYBP and MCM2 in CCA tissues was examined with The Cancer Genome Atlas (TCGA) database (https://cancergenome.nih.gov/). By adopting thresholds of false discovery rate (FDR) < 0.05 and |fold change| ≥ 1.5, differentially expressed genes (DEGs) between CACYBP-depleted and control HUCCT1 cells were chosen. Kaplan-Meier (KM) curves were plotted, which indicated correlation between CACYBP expression and patient survival rate. This work also conducted Pearson correlation for determining the relation of CACYBP with MCM2 expression (http://gepia2.cancer-pku.cn/#correlation).

### Cell culture and treatment

All cells, including four CCA cell lines (HUCCT1, HCCC-9810) together with healthy HIBEC cells employed in this work were provided by the cell bank of Chinese Scientific Academy (Shanghai, China). Cells were cultured in RPMI-1640 medium (Gibco, USA) that contained 1% penicillin/streptomycin (PS) as well as 10% fetal bovine serum (FBS) (Gibco, USA) and incubated with 5% CO_2_ under 37°C. Cycloheximide (CHX) (Sigma-Aldrich, USA) and proteasome inhibitor MG-132 (Sigma-Aldrich) were used after lentivirus transfection at 50 mg/mL and 10 μmol/L for 6 h, respectively. The Wnt/β-catenin inhibitor C59 (20 μmol/L, Cat No. HY-15659, MCE, China) was used for a 24-h period after lentivirus transfection.

### Lentivirus vector construction, package as well as cell transfection

For constructing the CACYBP knockdown, Yibeirui Technology (Shanghai, China) was responsible for preparing three short hairpin RNA (shRNA) sequences targeting human CACYBP gene (shCACYBP). BR-V108 plasmid (Yibeirui, China) was utilized to generate shCACYBP sequences. Lentiviral vector for MCM2 overexpression was generated by LV-013 plasmid (Yibeirui). In line with specific protocols, pMD2.G plasmid was co-transfected with pSPAX2 (Qiagen, China) plasmid in 293T cells to prepare shCACYBP and MCM2 lentiviruses. Scramble sequences and empty vector were the negative controls (NCs) for shCACYBP and MCM2, respectively. Lentivirus of shCACYBP and MCM2 were transfected into CCA cells with lipofectamine 2000 (Thermo Fisher, USA) at multiplicity of infection (MOI) = 20. Real-time quantitative (qPCR) was carried out to measure transfection efficiency. Target and shRNA sequences mentioned above are shown in Suppl. Table S1. HA tagged Ub(WT), Ub(K63) and Ub(K48) were transfected in to HUCCT1 cells, and further immunocoprecipitation (IP) was performed.

### qPCR

This work utilized TRIzol reagent (Sigma-Aldrich) for extracting total cellular RNA in line with specific instructions. Besides, Nanodrop 100 (Thermo Fisher) was adopted for quantifying the extracted RNA, which was synthesized into cDNA template with Hiscript QRT supermix (Vazyme, China). Thereafter, by adopting the Biosystems 7500 Sequence Detection system, 10 μL volume of qPCR reaction was carried out using SYBR Green master mix kit (Vazyme), with GAPDH being the endogenous control. Relative gene levels were determined by 2^−ΔΔCt^ approach. Suppl. Table S2 displays primers utilized in qPCR.

### Western-blot (WB) assay

The pre-chilled radioimmunoprecipitation (RIPA) lysis was added to collect the treated cells, then total proteins were determined using BCA Protein Assay Kit (HyClone-Pierce, USA). Later, 10% sodium dodecyl sulfate polyacrylamide gel electrophoresis (SDS-PAGE) (Invitrogen, USA) was conducted to separate whole-cell lysates, following by transfer on polyvinylidene difluoride (PVDF) membranes. The 5% skim milk was added to block membranes for a 1-h period, followed by overnight primary antibody incubation. Finally, secondary HRP-conjugated antibody was added for incubation for 2 h. Enhanced chemiluminescence (ECL) (Millipore) was utilized for determining relative protein expression. Antibodies used in western blot are listed in Suppl. Table S3.

### Cell counting assay

This assay was carried out for assessing the viability of treated cells. Briefly, 2000 cells were inoculated into the 96-well plates prior to lentivirus transfection; at 24 h later, C59 was added to treat transfected cells for a 24-h period. Subsequently, cell number was determined with the Celigo image cytometer (Nexcelom Bioscience) for five days consecutively. The cell proliferation curve was constructed based on obtained cell counts.

### Determination of cell apoptosis and cycle

Flow cytometry (FCM) was conducted to determine alterations of cell apoptosis and cycle. HUCCT1 and HCCC-9810 cells (1 × 106/well) were inoculated in the 6-well plates, followed by 24-h lentivirus transfection after reaching 70% density. After 24-h C59 treatment, cells were harvested and re-suspended before annexin V-APC staining (eBioscience, USA) for cell apoptosis assays. Similarly, cells were collected after treatment and fixed by 70% ethyl alcohol, followed by staining using the mixture of propidium iodide (PI) (Sigma-Aldrich) and RNase A (TaKaRa, China). A flow cytometer (Millipore, USA) was finally adopted for determining apoptotic cell proportion and cell distribution in diverse cell cycle phases.

### Determination of cell migration

Scratch and Transwell assays were conducted for evaluating cell migration. For wound-healing assays, this work inoculated HUCCT1 and HCCC-9810 cells (5 × 104/well) in 96-well plates after reaching 90% confluency. Following treatment of lentivirus infection, a scratch was generated at the bottom center of the 96-well plate, later, the 0.5% FBS-containing medium was added for cell culture. Cell migratory distance was determined at different time points in a cellomics (Thermo Fisher), and the migratory distance-to-initial distance ratio was calculated to determine cell migration rate.

In transwell assays, HUCCT1 and HCCC-9810 cells were trypsinized after transfection using the transwell kit (Corning, USA), re-suspended within non-FBS medium (100 μL), followed by addition into top chamber. Bottom chambers were then supplemented with 600 μL of 30% FBS, and top chamber that contained cells was transferred to bottom chamber. At 24-h post-incubation, a cotton tip was utilized to remove non-migratory cells on lower membrane surface, whereas Giemsa dye was added to stain migratory cells for a 5-min period. A fluorescence microscope was employed for calculating migratory cell number.

### Xenograft mouse model

Animal studies conducted for the purposes of this work gained approval from Ethics Committee of Shenzhen People’s Hospital. Mouse sufferings were maximally decreased by every effort.

Four-week-old BALB/c female nude mice (*n* = 20; Beijing Vital River Laboratory Animal Technology Co., Ltd., China; Approval No. SCXK 2016-0006) were raised under the specific-pathogen-free (SPF) condition. Animals were randomly separated as two groups, i.e., shCtrl and shCACYBP (*n* = 10 animals/group). Then, HUCCT1 cells at 1 × 10^7^ with or without CACYBP knockdown were subcutaneously injected in the flank area for tumorigenicity. We monitored tumor size every week, then determined tumor volume below: V = π/6 × L × W^2, in which L stands for vertical width whereas W indicates width at the widest position. Each mouse was injected with pentobarbital sodium (Merck, USA) for euthanasia on day 32, according to a previous study [38]. Later, we dissected tumor tissue and measured its weight, followed by photographing and tumor recording. Tumor tissue was later stained with H&E and Ki-67.

### Cell viability assay

The CCK-8 assay kit (MYBiotech, China) was utilized for determining cell viability. In brief, this work inoculated HUCCT1 and HCCC-9810 cells (3 × 103/well) in 96-well plates. Following infection and C59 treatment, all wells were introduced with CCK-8 solution (10 μL), followed by 4-h incubation. Absorbance (OD) values of cells were determined in the microplate reader (Thermo Fisher) at 450 nm. Cell viability curves were plotted using OD450 values.

### Statistical analysis

GraphPad Prism 8.04 software was utilized for statistical analysis. Data were represented by mean ± standard deviation (SD). Two-tailed Student’s *t*-test was conducted to analyze differences between two groups, with *p* < 0.05 indicating statistical significance. A log-rank test was adopted for calculating survival difference of Kaplan-Meier (KM) curves. Any commercialized kits, reagents, instruments, software, antibodies, etc., used in the research, shall be provided with their full name, along with the information of the manufacturers/suppliers/software details (Name, City, Province/State, Country).

## Results

### Overexpression of CACYBP within human CCA tissue

**Figure 1:**
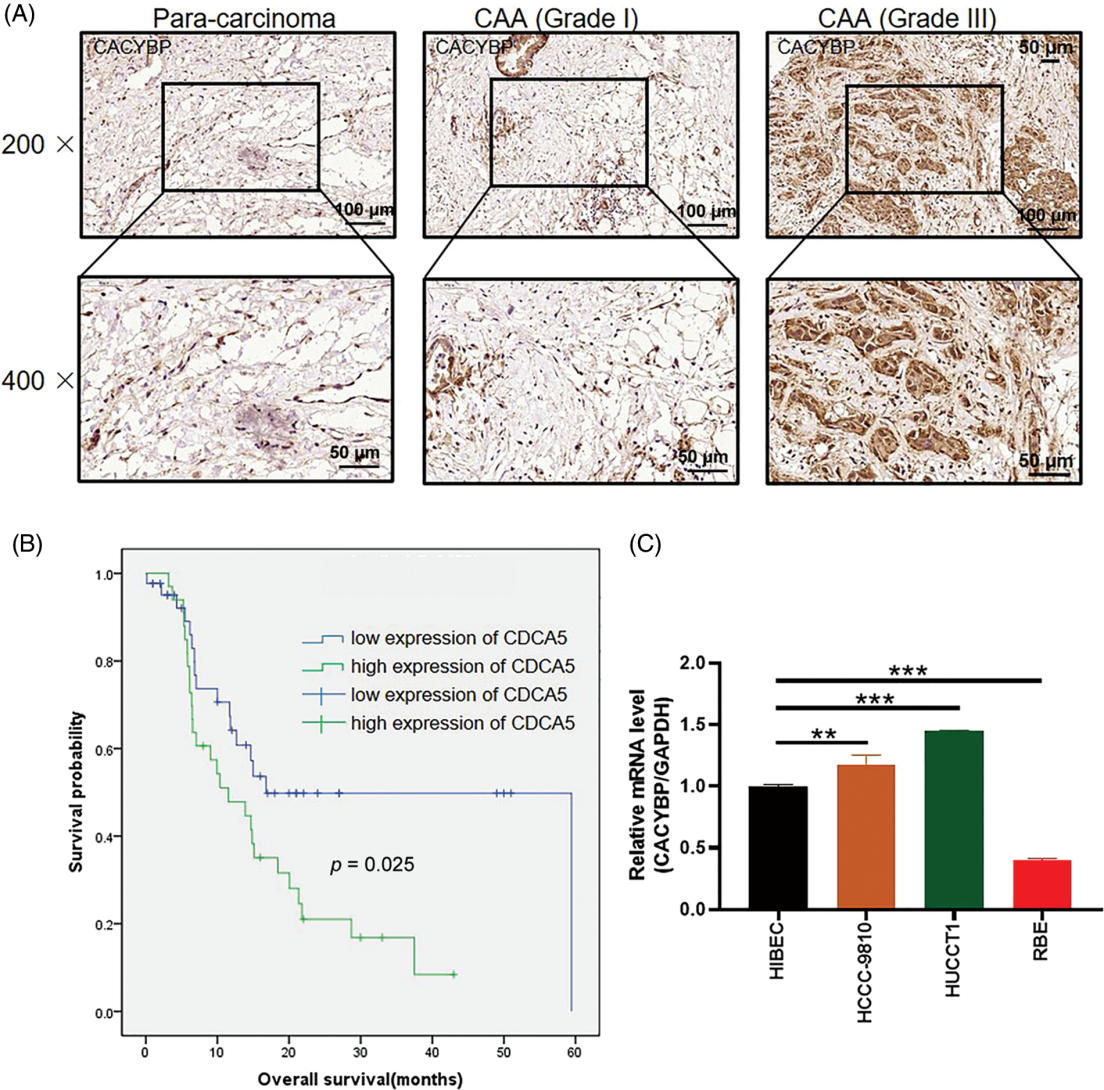
Calcyclin-binding protein (CACYBP) up-regulation in cholangiocarcinoma (CCA) cells. (A) Typical images showing CACYBP levels in CCA and non-carcinoma tissues based on immunohistochemistry. (B) Kaplan-Meier curves indicated a significant relation of CACYBP level with overall survival (OS) of CCA cells. CACYBP high expression: 35 samples; CACYBP low expression: 44 samples. (C) Relative mRNA level of CACYBP in four CCA cell lines and control cell line as determined by qPCR.

**Supplementary Figure 1:**
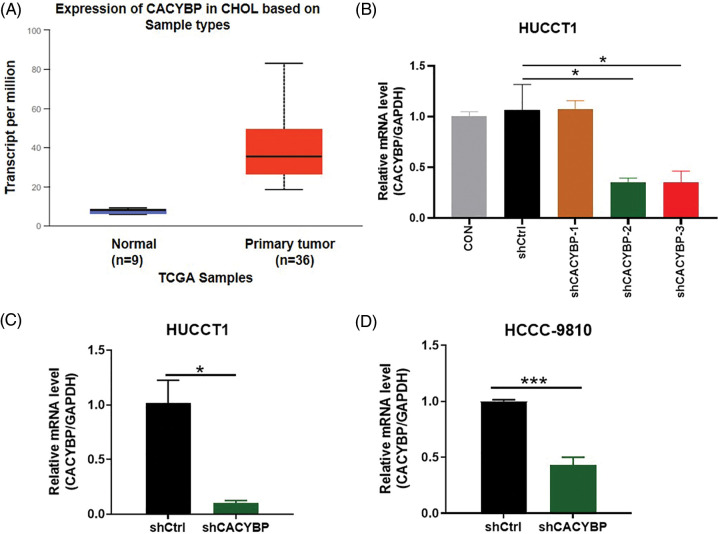
Transfection efficacy and short hairpin RNA (shRNA) knockdown efficiency. (A) Calcyclin-binding protein (CACYBP) expression level in cholangiocarcinoma (CCA) samples in TCGA-CCA (*n* = 36) *vs*. control samples (*n* = 9). (B) Transfection efficacy of three shCACYBP lentivirus determined by qPCR analysis. (C) qPCR analysis of knockdown of CACYBP in HUCCT1 and (D) HCCC-9810. Data are represented by mean ± standard deviation. *p < 0.05, ***p < 0.001.

For identifying CACYBP’s function for CCA development, CACYBP levels within TCGA-CCA (*n* = 36) were analyzed. CACYBP showed high expression within CCA in comparison with non-carcinoma tissue (*n* = 9) (Suppl. Fig. S1A). In addition, CACYBP levels within CCA (*n* = 79) and normal para-carcinoma tissues (*n* = 26) of human tissue chip was observed using IHC analysis. As shown in IHC staining representative images, CACYBP was found highly expressed within CCA in comparison with normal tissues (Fig. 1A). Furthermore, CACYBP expression was significant difference between CCA (44.3%) and non-carcinoma tissues (3.8%) (*p* < 0.001) (Table 1). Moreover, by analyzing relationship of CACYBP level with tumor features in CCA patients, CACYBP overexpression showed positive relation to T stage, tumor grade and recurrence of state (*p* < 0.05) (Table 2), as confirmed through Pearson correlation analysis (Table 3). Accordingly, based on KM analysis, CACYBP up-regulation predicted the dismal prognosis (Fig. 1B). Consistently, CACYBP was also found expressed at higher levels in four CCA cells compared with healthy HIBEC cells (Fig. 1C). Collectively, the above findings indicated CACYBP up-regulation within human CCA tissues, which predicted CCA progression.

**Table 1:** Expression levels within cholangiocarcinoma and non-carcinoma tissues as determined by IHC

CACYBP level	Tumor tissue		Non-carcinoma tissue		*p* value
	Case	Percentage	Case	Percentage	
Low	44	55.7%	25	96.2%	<0.001
High	35	44.3%	1	3.8%	

**Table 2:** Relationship between calcyclin-binding protein (CACYBP) level with tumor features of cholangiocarcinoma cases

Feature	Case (*n* = 79)	CACYBP level		*p* value
		Low	High	
Age(years)				0.515
≤56	40	24	16	
>56	38	20	18	
Gender				0.600
Male	41	24	17	
Female	38	20	18	
Grade				<0.01
I	5	5	0	
II	57	35	22	
III	17	4	13	
Lymphatic metastasis (N)				0.622
N0	52	30	22	
N1	27	14	13	
T stage				0.014
T1-T2	60	38	22	
T3-T4	18	5	13	
Tumor size				0.240
≤3cm	42	26	16	
>3cm	37	18	19	
Stage				0.074
1-2	43	28	15	
3-4	46	16	20	
Lymphoid positive number				0.748
=0	52	30	22	
>0	26	14	12	
Recurrence of stage				<0.001
no	29	24	5	
yes	47	18	28	

**Table 3:** Pearson correlation coefficients of calcyclin-binding protein (CACYBP) level with tumor features of cholangiocarcinoma cases

Feature	n	*R* value	*p* value
Grade	79	0.386	<0.001
Recurrence of stage	76	0.415	<0.001

### CACYBP depletion impaired oncogenic features of CCA cells

**Figure 2:**
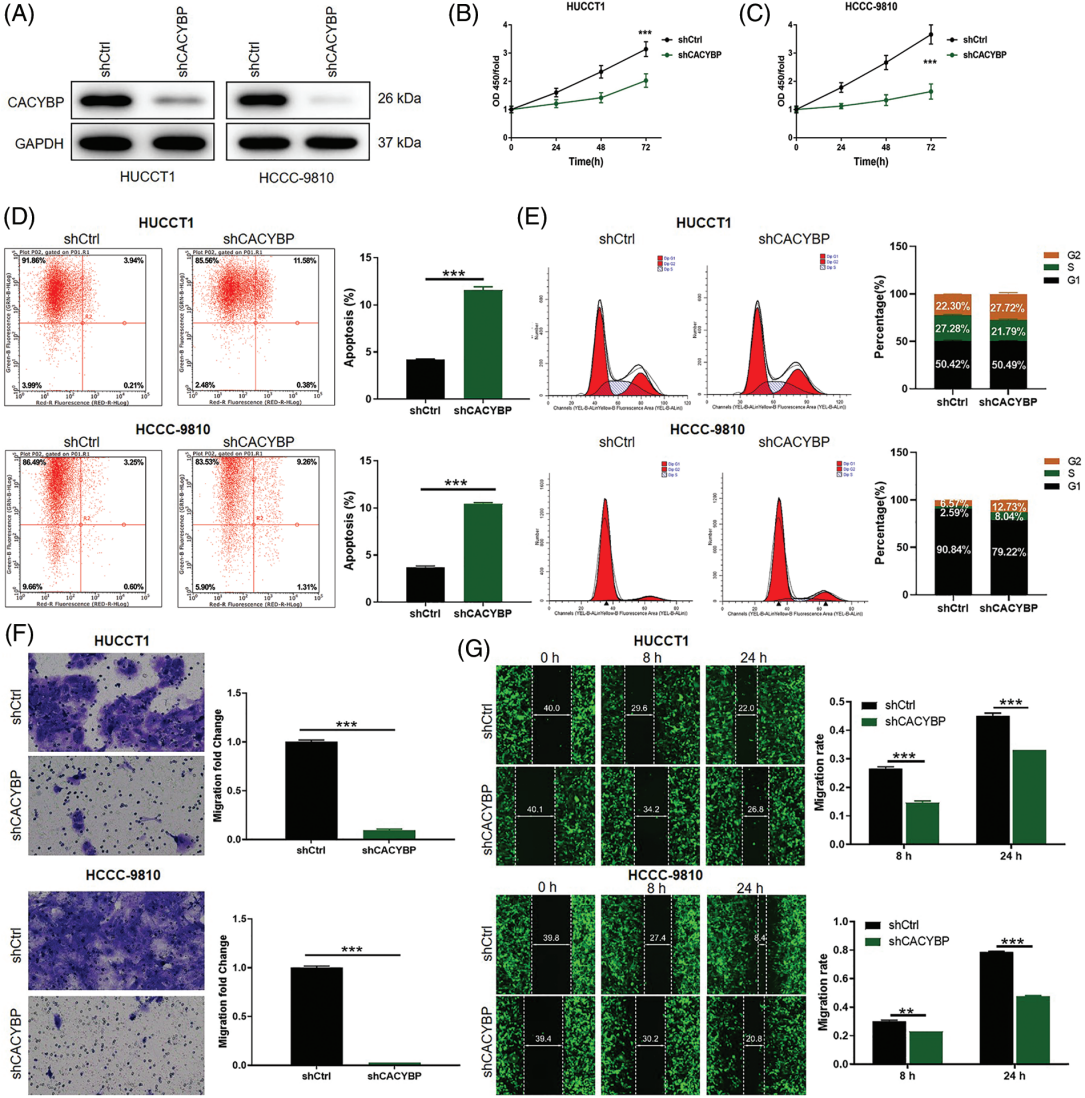
Calcyclin-binding protein (CACYBP) silencing impaired oncogenic features in cholangiocarcinoma (CCA) cell lines. (A) CACYBP protein levels in HUCCT1 and HCCC-9810 cell lines transfected with or without shCACYBP lentivirus as measured through WB assays, with GAPDH being an internal reference. (B) Viability of HUCCT1 and HCCC-9810 cells after CACYBP silencing as determined by Celigo cell count assay. (C) Changes in cell apoptosis and (D) cell cycle in HUCCT1 and HCCC-9810 cells after CACYBP silencing as determined through FCM. (E) HUCCT1 and HCCC-9810 cell migration after CACYBP silencing as determined through transwell and (F) scratch assays. Results are represented by mean ± standard deviation. **p < 0.01, ***p < 0.001.

To elucidate CACYBP’s function during CCA occurrence, stable CACYBP knockdown HUCCT1 and HCCC-9810 cell lines were generated. Specifically, the interference sequence shCACYBP-2 was selected to knockdown CACYBP in HUCCT1 and HCCC-9810 cells; qPCR and WB assays were conducted to verify gene knockdown (Suppl. Figs. S1B–S1D and Fig. 2A).

Considering the CACYBP knockdown cell model, changes in cell viability, apoptosis, migration and cell cycle distribution were analyzed. Cell viability was markedly suppressed in the shCACYBP group in comparison with shCtrl group for CCA cells (*p* < 0.01) (Figs. 2B and 2C). Additionally, CACYBP knockdown promoted apoptosis in HUCCT1 and HCCC-9810 in comparison with shCtrl (*p* < 0.001) (Fig. 2D). Furthermore, CACYBP knockdown halted HUCCT1 and HCCC-9810 cell cycle in G2 phase, and markedly declined HUCCT1 cells in S stage (*p* < 0.001) (Fig. 2E). Further, according to scratch and transwell assays, CACYBP knockdown dramatically decreased CCA cell migration, inconsistent with findings found in shCtrl (*p* < 0.01) (Figs. 2F and 2G). Collectively, CACYBP knockdown suppressed oncogenic features of malignant CCA cell growth and invasion and induced cell cycle arrest.

### CACYBP knockdown inhibited tumor formation in mice

**Figure 3:**
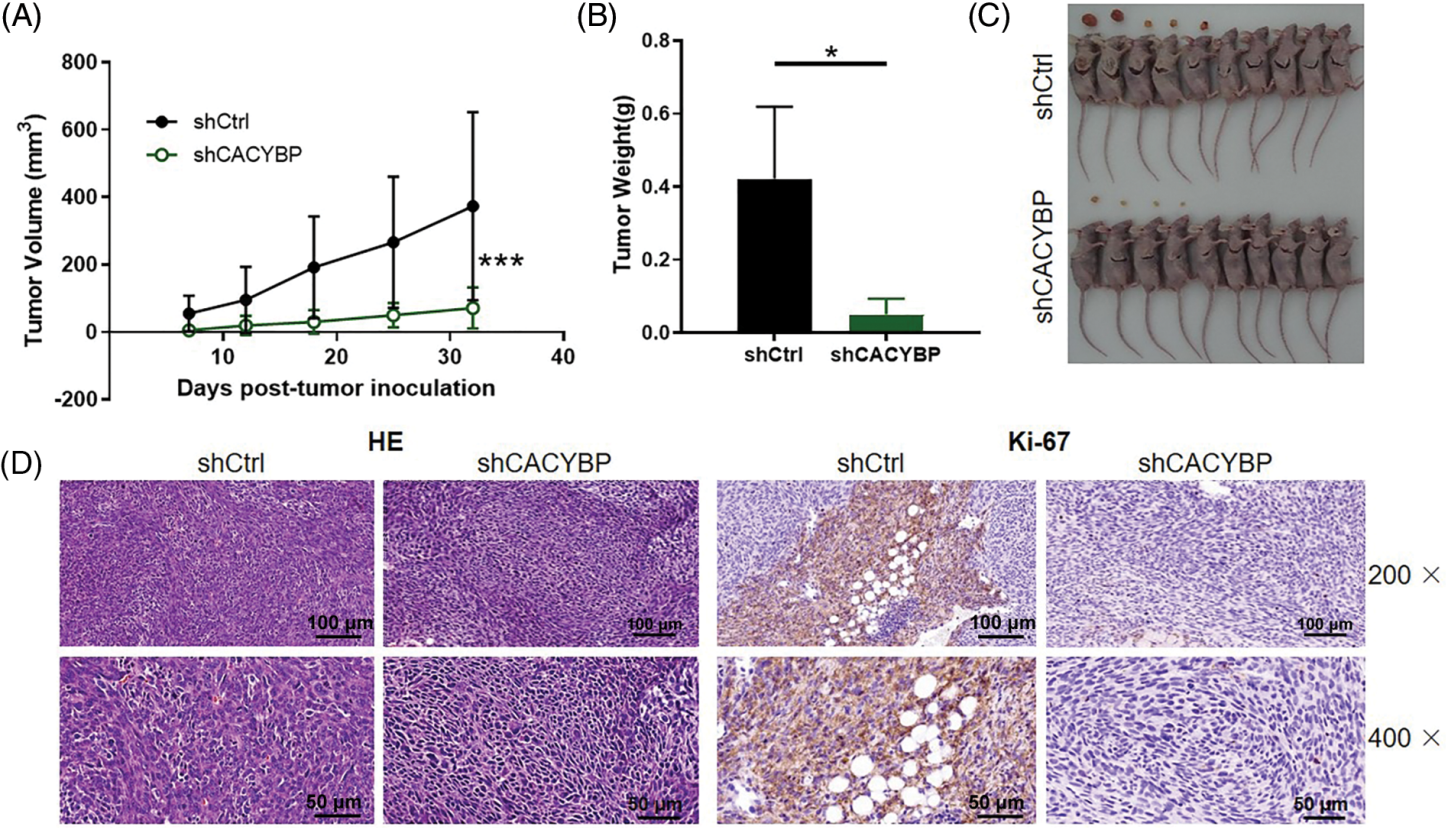
Calcyclin-binding protein (CACYBP) silencing inhibited tumorigenesis of mice. (A) Tumor growth curves and (B) tumor weight in mice given injection of CACYBP-silenced HUCCT1 cells and control cells. Tumor size was determined on days specified. (C) Photographs of mice and tumors isolated from mice. (D) H and E staining and (D) Ki-67 staining observations on tumor tissues in shCtrl (control) group and shCACYBP experimental group. Data are represented by mean ± standard deviation. *p < 0.05, ***p < 0.001.

Subsequently, this work conducted *in-vivo* assays for assessing CACYBP’s effect on tumor formation. CACYBP knockdown HUCCT11 cells were injected subcutaneously to construct a xenograft mice model. One week after inoculation, tumor size was measured until animals were sacrificed. CACYBP knockdown significantly attenuated tumor formation in mice (*p* < 0.01) (Fig. 3A). In agreement with tumor volume, tumor weight also showed a remarkable reduction when CACYBP was silenced (*p* < 0.05) (Figs. 3B and 3C). In addition, nodules in shCtrl and shCACYBP groups stained with H&E, thereby confirming their nature as tumor tissue (Fig. 3D). Subsequently, it was found that CACYBP silencing apparently suppressed the expression of Ki-67 in comparison with shCtrl (Fig. 3E). Collectively, CACYBP exerts an essential role in CCA progression.

### CACYBP silencing facilitated the degradation of MCM2 by inducing ubiquitination

**Figure 4:**
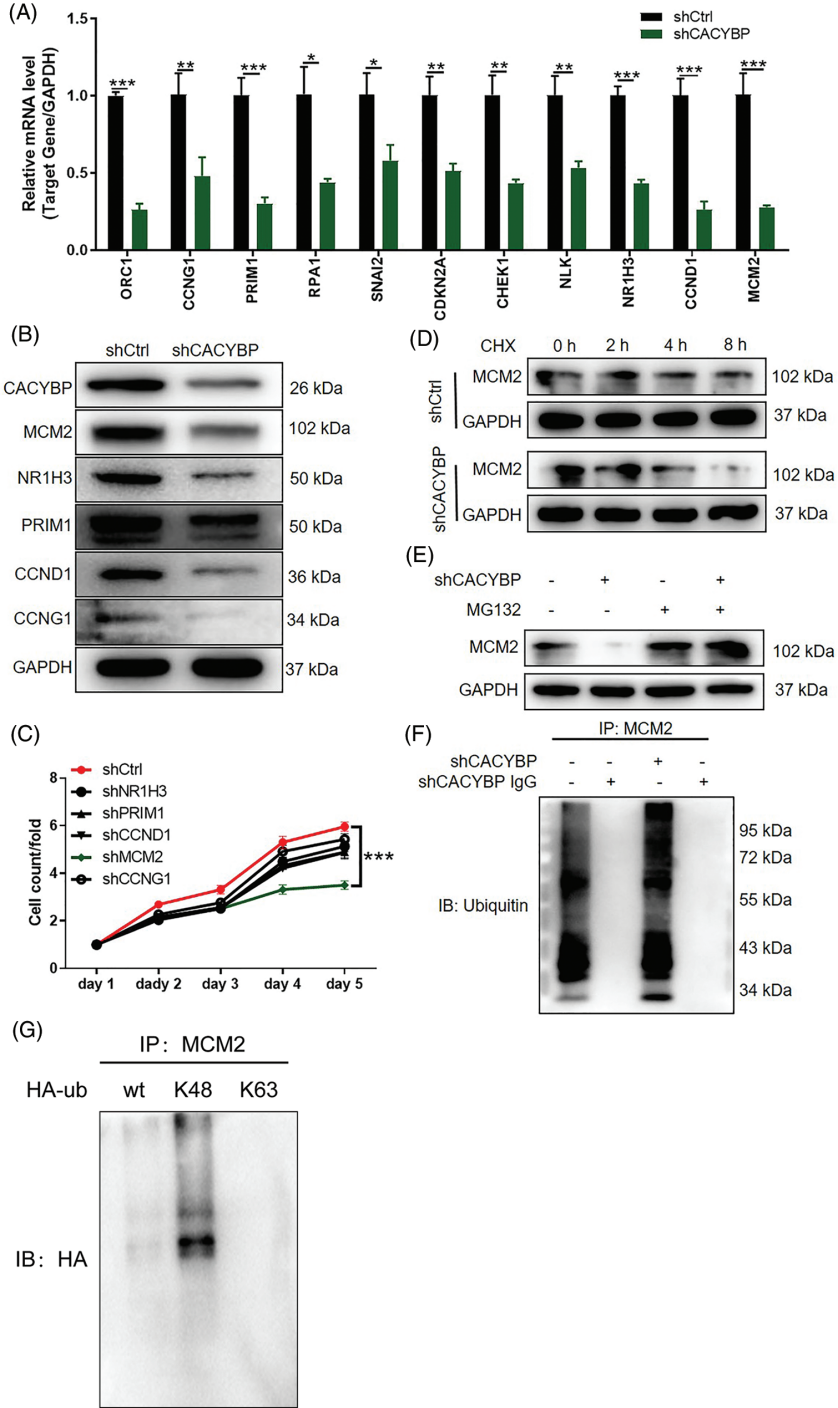
Calcyclin-binding protein (CACYBP) knockdown facilitated degradation of minichromosome maintenance complex component 2 (MCM2) by inducing ubiquitination. (A) Relative mRNA levels of the top eleven differentially expressed genes (DEGs) in HUCCT1 cells after CACYBP silencing as determined by qPCR analysis. (B) Relative protein levels of MCM2, NR1H3, PRIM1, CCND1 and CCNG1 in HUCCT1 cells after CACYBP silencing as determined by western blot assays. (C) Viability of HUCCT1 cells after knockdown of NR1H3, PRIM1, CCND1, CCNG1 and MCM2 as assessed by Celigo cell count assays at indicated times. (D) Relative protein level of MCM2 in CACYBP-knockdown HUCCT1 cells treated with cycloheximide (50 mg/mL). (E) Relative protein level of MCM2 in CACYBP-knockdown HUCCT1 cells exposed to 10 μmol/L proteasome inhibitor MG-132 treatment. (F) Ubiquitination of MCM2 immunoprecipitated by anti-MCM2 antibody. Data are represented by mean ± standard deviation. (G) Detection of K48 and K63 mediated ubiquitination of MCM2 in HUCCT1 cells. *p < 0.05, **p < 0.01, ***p < 0.001.

**Supplementary Figure 2:**
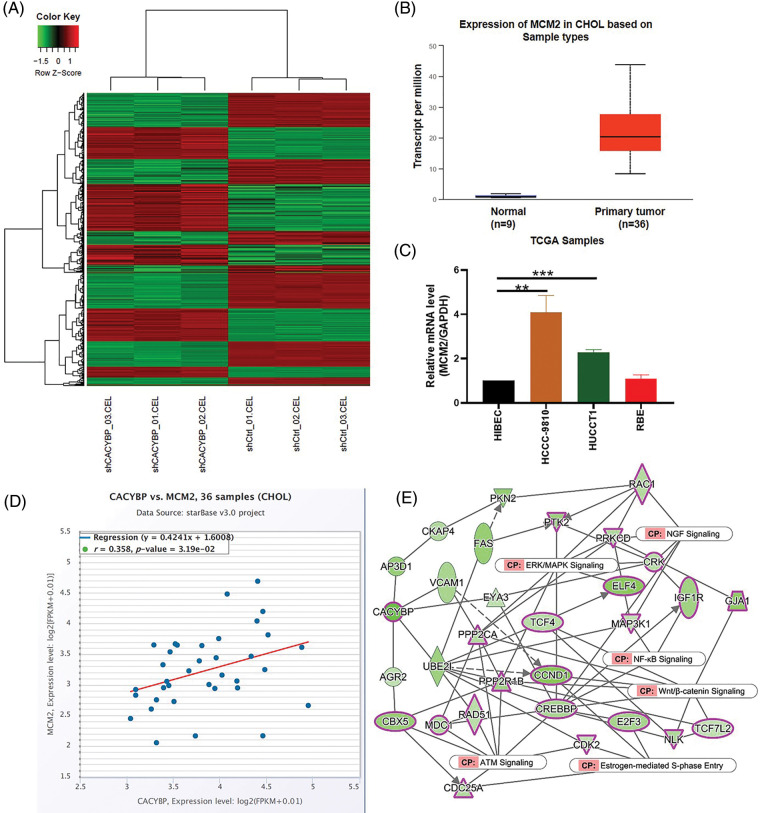
Screening of potential regulatory protein of calcyclin-binding protein (CACYBP). (A) Heatmap showing differentially expressed genes (DEGs) in CACYBP-silenced HUCCT1 compared with control cells as determined by hierarchical clustering analysis of IPA. (B) Minichromosome maintenance complex component 2 (MCM2) expression level in cholangiocarcinoma (CCA) samples in TCGA-CCA (*n* = 36) *vs*. control samples (*n* = 9). (C) Relative mRNA level of MCM2 in four CCA cell lines and control cells. (D) Pearson correlation analysis between CACYBP and MCM2. (E) IPA identified interaction between CACYBP and pathways of NGF signaling, estrogen-mediated S-phase entry, Wnt/β-catenin signaling, ERK/MAPK signaling, NF-KB signaling, ATM signaling. Data are represented by mean ± standard deviation. *p < 0.05, ***p < 0.001.

Further, to investigate the co-expression partners of CACYBP, DEGs between shCACYBP and shCtrl conditions were identified using microarray analysis. In total, 673 genes were found upregulated, whereas 689 genes were downregulated following CACYBP silencing (Fig. S2A). Next, the relative gene expression of top-ranked DEGs was validated by qPCR (*p* < 0.05) (Fig. 4A), and the downregulation of MCM2, NR1H3, PRIM1, CCND1 and CCNG1 upon CACYBP silencing was confirmed by western blot analysis (Fig. 4B). Among these proteins, MCM2 expression distinctly increased within CCA cells and tissues relative to controls (Figs. S2B and S2C). According to Pearson correlation analysis, CACYBP expression was positively correlated with MCM2 (Fig. S2D). Additionally, relative to shNR1H3, and shPRIM1, shCCND1 and shCCNG1 groups, cell viability of shMCM2 group was evidently inhibited (Fig. 4C). Therefore, it can be assumed that MCM2 might be a co-expression partner of CACYBP for CCA progression.

Ubiquitination has an important regulatory effect on cell survival, differentiation, cycle distribution, and others [17], and that CACYBP is reportedly involved in the regulation of protein stability [18]. Thus, we sought to explore the connection between CACYBP and ubiquitination of MCM2. As expected, a decrease in MCM2 stability was observed in CACYBP-silenced HUCCT11 cells upon CHX treatment (Fig. 4D). Notably, the effect of CACYBP knockdown on the stability of MCM2 was ameliorated by treatment with the proteasome inhibitor MG-132, which indicated that CACYBP may regulate MCM2 via the ubiquitin-proteasome system (UPS) (Fig. 4E). Thus, using a ubiquitination assay, it was shown that CACYBP silencing significantly increased MCM2 ubiquitination (Fig. 4F). And K48 of a Ub moiety is critical for mediating MCM2 ubiquitination, which mediates MCM2 protein degradation (Fig. 4G). Collectively, these results suggested that CACYBP knockdown induced ubiquitination of MCM2, thereby weakening its protein stability.

### CACYBP promoted CCA cell growth and migration but suppressed apoptosis by stabilizing MCM2

**Figure 5:**
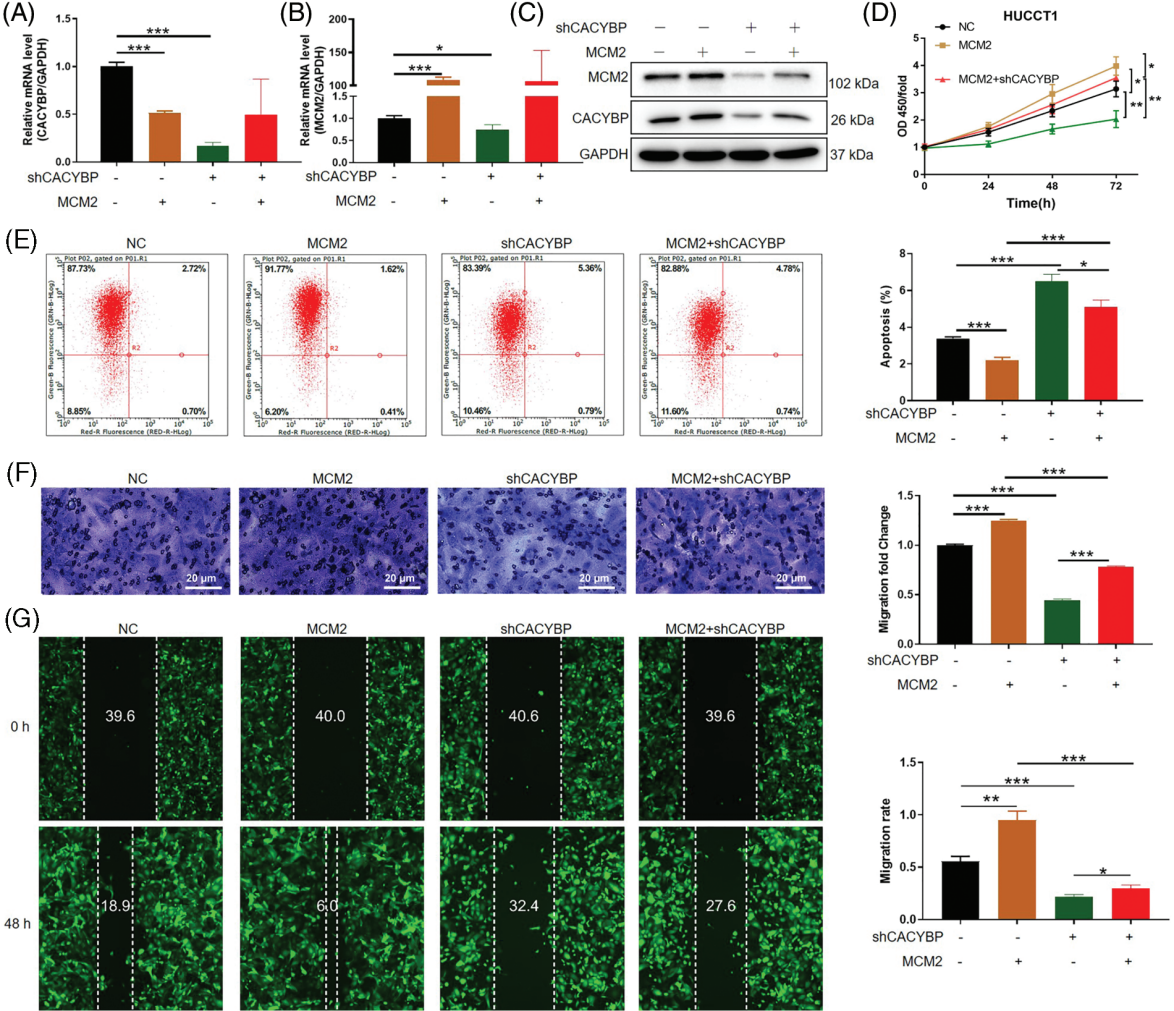
Calcyclin-binding protein (CACYBP) promoted cholangiocarcinoma (CCA) growth and migration, as well as suppressed apoptosis by stabilizing minichromosome maintenance complex component 2 (MCM2). (A and B) Relative MCM2 and CACYBP mRNA and (C) protein expression within HUCCT1 cell after transfection of shCACYBP, MCM2, and shCACYBP+MCM2 lentivirus. (D) Cell viability, (E) apoptosis and (F and G) migration as demonstrated through Celigo cell count assay, FCM, scratch and transwell assays, respectively. Images selected were representatives of from three separate assays. Results are represented by mean ± standard deviation. *p < 0.05, **p < 0.01, ***p < 0.001.

To confirm MCM2’s important effect on oncogenic function of CACYBP in CCA, MCM2 was overexpressed on the basis of CACYBP silencing in HUCCT11 cells. Based on the data of qPCR and western blot analysis, CACYBP and MCM2 were successfully downregulated and upregulated, respectively (Figs. 5A–5C). Moreover, downregulation of CACYBP led to a significant decrease in MCM2, while MCM2 overexpression did not affect transcription and translation of CACYBP, indicating that MCM2 could be considered a downstream gene to CACYBP. Subsequently, it was found that MCM2 overexpression led to increased cell viability, which impaired the suppression effect of shCACYBP on cell growth (Fig. 5D). Consistently, the increased cell apoptosis in the shCACYBP group was remarkably attenuated due to MCM2 overexpression (Fig. 5E). Similarly, cell migration inhibition due to CACYBP silencing was compromised as a consequence of MCM2 overexpression (Figs. 5F and 5G). Thus, these findings suggested that MCM2 acts as an essential downstream factor to CACYBP, which contributes to CCA progression.

### Wnt/β-catenin pathway activation is required for MCM2 regulation within CCA cells

**Figure 6:**
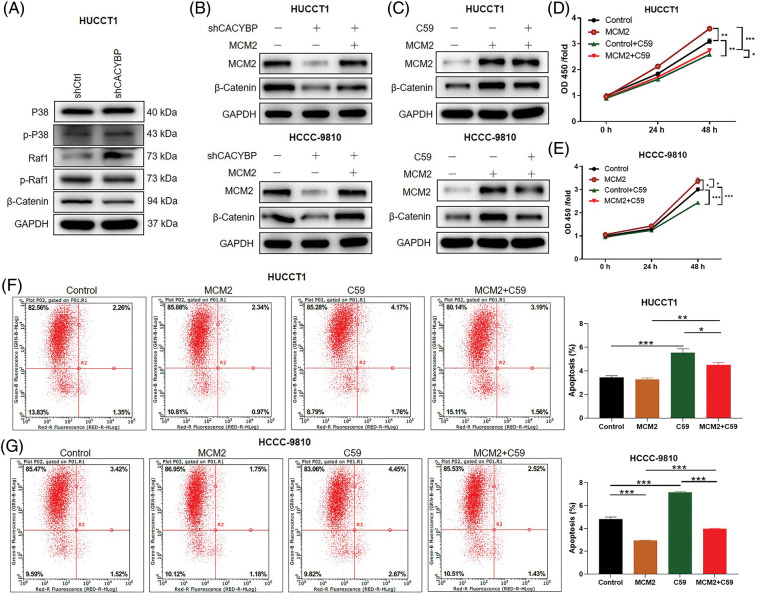
Wnt/β-catenin pathway is necessary for minichromosome maintenance complex component 2 (MCM2) regulation in cholangiocarcinoma (CCA) cells. (A) Several regulatory signaling pathways were determined by western blot assays in HUCCT1 cells after calcyclin-binding protein (CACYBP) silencing. (B) Relative protein levels of MCM2 and β-catenin in HUCCT1 and HCCC-9810 cells infected with shCACYBP or shCACYBP+MCM2 lentivirus. (C) Relative protein levels of MCM2 and β-catenin within HUCCT1 and HCCC-9810 cells treated with MCM2 lentiviral transfection or C59 inhibitor (20 μmol/L) combined with MCM2 lentivirus transfection. (D) HUCCT1 and (E) HCCC-9810 cell viability as determined through CCK-8 assays. (F) HUCCT1 and (G) HCCC-9810 cell apoptosis as measured through FCM. Data are represented by mean ± standard deviation *p < 0.05, **p < 0.01, ***p < 0.001.

Finally, based on the analysis of IPA interaction network, it was revealed that DEGs were significantly enriched into Wnt/β-catenin pathway (Suppl. Fig. S2E). According to western blot assay, β-catenin levels were found decreased in HUCCT11 cells upon CACYBP silencing, while the levels of p-P38 and Raf1 were upregulated (Fig. 6A). Moreover, CACYBP silencing inhibited β-catenin levels within HUCCT11 and HCCC-9810 cells, which was alleviated by MCM2 up-regulation (Fig. 6B).

Furthermore, to elucidate effect of Wnt/β-catenin pathway on CCA, Wnt/β-catenin signaling inhibitor C59 was used. In the presence of C59, the promoting effect resulting from MCM2 upregulation on β-catenin protein levels was partly reversed in two CCA cell lines (Fig. 6C). Furthermore, suppressing Wnt/β-catenin pathway significantly attenuated cell viability promoted by MCM2 upregulation in both CCA cell lines (Figs. 6D and 6E). Consistently, inhibiting Wnt/β-catenin pathway alos reverted apoptosis decrease induced by MCM2 overexpression (Figs. 6F and 6G). Altogether, Wnt/β-catenin signaling pathway may be the possible downstream factor of MCM2 for CCA development.

## Discussion

CCA is a type of highly aggressive and heterogenous epithelial cancer, which poses a serious threat to patients’ health [19]. Previous studies have suggested an increasing incidence of CCA, and its silent presentation further aggravates its alarming mortality [20]. Over the last few decades, five-year survival and tumor recurrence rates after tumor resection are still greatly discouraging due to limited awareness regarding CCA occurrence and development [21,22]. Therefore, unveiling the pathogenesis of CCA is urgently needed to provide patients with new therapeutic options to improve patient outcomes. Herein, the overexpression of CACYBP and MCM2 in CCA was observed, which predicted dismal patient prognosis. In addition, CACYBP silencing inhibited oncogenic features in CCA cell lines and suppressed tumor formation in mice. Moreover, CACYBP inhibited ubiquitination of MCM2, thereby enhancing the stability of MCM2 to facilitate CCA progression. Interestingly, Wnt/β-catenin pathway may be the downstream factor to MCM2 in CCA development. Collectively, the results discussed herein evidentiated that CACYBP acts as a promising prognostic biomarker.

Furthermore, previous articles suggest that CACYBP promotes the development of several cancer types. In hepatocellular carcinoma, CACYBP could regulate cancer cell growth, apoptosis and cell cycle, thereby accelerating tumor progression, and CACYBP overexpression also predicted a poor prognosis [23]. However, the function of CACYBP in CCA remains unknown. Herein, CACYBP showed high expression within human CCA in comparison with non-carcinoma tissues, in line with the data from TCGA. Moreover, CACYBP overexpression was markedly related to the characteristics of tumor grade and recurrence of state. Additionally, CACYBP silencing was able to suppress CCA cell growth and migration, but induced cycle arrest *in vitro*. Notably, CACYBP was also required for tumor formation in mice. Taken together, CACYBP had a promoting effect on CCA. However, the underlying mechanism of CACYBP contributing to CCA progression needs to be further elucidated.

MCM2 is one subunit of helicase complex that exerts a crucial effect on regulating DNA replication [24,25]. During S phase, MCM complex triggers DNA replication initiation and restricts the chromosome replication times upon cell cycle kinases [26,27]. Mutation in MCM complex reportedly results in chromosome loss and DNA damage in yeast [28]. Similarly, aberrant expression of MCM2 in human cau cause development of oral cancer [29], BC [30] and GC [31], suggesting that MCM2 is the potential biomarker used to predict the prognosis of certain tumors. Nonetheless, MCM2’s effect on CCA is still largely unknown. Herein, MCM2 showed high expression within CCA tissues, which was positively correlated with CACYBP overexpression. Further experiments indicated that MCM2 knockdown apparently impaired CCA cell viability, suggesting that MCM2 might be a co-expression partner of CACYBP.

Ubiquitination is an imperative post-translation process in eukaryotes, and CACYBP is recently suggested to suppress glioblastoma invasion and migration by modulating cytoplasmic p27 degradation and ubiquitination [32]. Herein, we thus confirmed the regulatory function of CACYBP on the stability of MCM2. It was also found that targeting CACYBP not only reduces the expression of MCM2, NR1H3, PRIM1, CCND1 and CCNG1, but also impacts the stability of CACYBP for MCM2 at the protein level. Accordingly, the upregulation of MCM2 partly reversed the anti-apoptotic and inhibition effect of CACYBP in CCA cell proliferation. Altogether, these findings emphasized the importance of MCM2 in CCA progression facilitated by CACYBP. However, the specific mechanism underlying the CACYBP-mediated inhibition of MCM2 ubiquitination was not illustrated in this work.

Finally, IPA results clarified that CACYBP interacted with Wnt/β-catenin pathway, whereas relationship of MCM2 with Wnt/β-catenin pathway was less studied. Wnt/β-catenin pathway is highly conserved and controls cellular proliferation and differentiation [33]. Notably, a growing amount of evidence has indicated that a dysfunctional Wnt/β-catenin pathway may be related to tumorigenesis, including CCA [34,35]. Recently, this pathway was shown to induce multidrug resistance (MDR) through P-glycoprotein efflux pump level in CCA cells, thus indicating its potential as the anti-CCA pharmacological target [36]. Herein, suppressing Wnt/β-catenin signaling and CACYBP silencing were observed in CCA. However, when using Wnt/β-catenin inhibitor C59, promoting function of MCM2 upregulation on β-catenin within CCA cells was abolished. Moreover, inhibiting Wnt/β-catenin signaling alleviated the decrease in apoptosis induced by MCM2 overexpression. Therefore, Wnt/β-catenin signaling pathway may be the possible downstream factor to MCM2 in CCA development.

## Conclusion

Collectively, CACYBP was identified to be the novel gene involved in CCA growth, and it was the potential anti-CCA pharmacological target. Especially, CACYBP may contribute to the development of CCA by stabilizing MCM2 protein, which can then activate Wnt/β-catenin pathway. However, it should be elucidated whether CACYBP directly regulates the protein stability of MCM2 to promote CCA growth.

## Data Availability

All data obtained in the present work can be obtained from this manuscript and the supplementary data files.

## Abbreviations

CCA: Cholangiocarcinoma
CACYBP: Calcyclin-binding protein
IHC: Immunohistochemical

## Supplementary Materials

**Table S1:** Target and short hairpin RNA (shRNA) nucleotide sequences

Gene	No.	Target sequence (5′-3′)	shRNA sequences (5′-3′)
CACYBP	CACYBP-1	AGCCAAAGGAGACACGGAATT	ccgg AGCCAAAGGAGACACGGAATT ctcgag AATTCCGTGTCTCCTTTGGCT tttttg
CACYBP	CACYBP-2	ATGATATGAAGCGAACCATTA	ccgg ATGATATGAAGCGAACCATTA ctcgag TAATGGTTCGCTTCATATCAT tttttg
CACYBP	CACYBP-3	GAATCTAAATGGGAAGAGTTA	ccgg GAATCTAAATGGGAAGAGTTA ctcgag TAACTCTTCCCATTTAGATTC tttttg

**Table S2:** Primers utilized for qPCR

Primer name	Forward sequence (5′-3′)	Reverse sequence (5′-3′)
CACYBP	ACAGATCCTAGTGAGGGATTGATG	TCCGTGTCTCCTTTGGCTTG
MCM2	CCAATGGCTTCCCTGTCTT	TCATCGGTCAGTTCCCCTAC
ORC1	AAAAGCCCAGAATGAAGC	TTACCTAGAAACCGAAGC
CCNG1	CAGAATGACTGCAAGACTAAGGG	AGTCAGTTGCCAATGGGACA
PRIM1	GCGCTCAATGGAGACGTTTG	CCGTAGTTGAGCCAGCGATA
RPA1	TTAATGCTGCGCTTCGCTTG	GCTGCAAACACTAGGAAGAAGG
SNAI2	AAAGCCAAACTACAGCGAACTG	TGGTATGACAGGCATGGAGTAA
CDKN2A	GCGAGTGAGGGTTTTCGTGGTTCA	CCTGGTCTTCTAGGAAGCGGCTGC
CHEK1	TTGGCTTGGCAACAGTATTTCG	CCAGCGAGCATTGCAGTAAGT
NLK	ACTCAGTATTATCGGGCTCCAG	CATCCCACAGACCAGATGTCA
NR1H3	CGCAAATGCCGTCAGG	TGCCGCTTCAGTTTCTTCA
CCND1	GAGGAAGAGGAGGAGGAGGA	TCATCGGTCAGTTCCCCTAC
GAPDH	TGACTTCAACAGCGACACCCA	CACCCTGTTGCTGTAGCCAAA

**Table S3:** Antibodies utilized for Western-blot assay

Assay	Primary antibody	Size/kDa	Diluted multiples	Source	Company	Catalog No.
WB	CACYBP	26	1:2000	Rabbit	Abcam	Ab171972
	MCM2	102	1:2000	Rabbit	Proteintech	10513-1-AP
	CCND1	36	1:3000	Rabbit	Abcam	Ab134175
	CCNG1	34	1:1000	Rabbit	Proteintech	10897-1-AP
	NR1H3	50	1:1000	Rabbit	Affbiotech	DF6864
	PRIM1	50	1:1000	Rabbit	Abcam	Ab137917
	β-Catenin	94	1:2000	Rabbit	Abcam	Ab16051
	GAPDH	37	1:3000	Rabbit	Bioworld	AP0063
Secondary antibody	Diluted multiples				Company	Catalog No.
Goat Anti-Rabbit	1:3000				Beyotime	A0208
Goat Anti-Mouse	1:3000				Beyotime	A0216

## References

[1] Rizvi, S., Gores, G. J. (2013). Pathogenesis, diagnosis, and management of cholangiocarcinoma. Gastroenterology*,* 145 *(*6*),* 1215–1229. 10.1053/j.gastro.2013.10.013; 24140396 PMC3862291

[2] DeOliveira, M. L., Cunningham, S. C., Cameron, J. L., Kamangar, F., Winter, J. M. et al. (2007). Cholangiocarcinoma: Thirty-one-year experience with 564 patients at a single institution. Annals of Surgery*,* 245 *(*5*),* 755–762. 10.1097/01.sla.0000251366.62632.d3; 17457168 PMC1877058

[3] Everhart, J. E., Ruhl, C. E. (2009). Burden of digestive diseases in the United States Part III: Liver, biliary tract, and pancreas. Gastroenterology*,* 136 *(*4*),* 1134–1144. 10.1053/j.gastro.2009.02.038; 19245868

[4] Khan, S. A., Davidson, B. R., Goldin, R. D., Heaton, N., Karani, J. et al. (2012). Guidelines for the diagnosis and treatment of cholangiocarcinoma: An update. Gut*,* 61 *(*12*),* 1657–1669. 10.1136/gutjnl-2011-301748; 22895392

[5] Jarnagin, W. R., Fong, Y., DeMatteo, R. P., Gonen, M., Burke, E. C. et al. (2001). Staging, resectability, and outcome in 225 patients with hilar cholangiocarcinoma. Annals of Surgery*,* 234 *(*4*),* 507–517. 10.1097/00000658-200110000-00010; 11573044 PMC1422074

[6] Yoo, C., Kim, K. P., Jeong, J. H., Kim, I., Kang, M. J. et al. (2021). Liposomal irinotecan plus fluorouracil and leucovorin versus fluorouracil and leucovorin for metastatic biliary tract cancer after progression on gemcitabine plus cisplatin (NIFTY): A multicentre, open-label, randomised, phase 2b study. Lancet Oncolog*,* 22 *(*11*),* 1560–1572. 10.1016/S1470-2045(21)00486-1; 34656226

[7] Rizvi, S., Yamada, D., Hirsova, P., Bronk, S. F., Werneburg, N. W. et al. (2016). A hippo and fibroblast growth factor receptor autocrine pathway in cholangiocarcinoma. Journal of Biological Chemistry*,* 291 *(*15*),* 8031–8047. 10.1074/jbc.M115.698472; 26826125 PMC4825008

[8] Sato, K., Glaser, S., Alvaro, D., Meng, F., Francis, H. et al. (2020). Cholangiocarcinoma: Novel therapeutic targets. Expert Opinion on Therapeutic Targets*,* 24 *(*4*),* 345–357. 10.1080/14728222.2020.1733528; 32077341 PMC7129482

[9] Matsuzawa S. I., Reed J. C. (2001). Siah-1, SIP, and Ebi collaborate in a novel pathway for beta-catenin degradation linked to p53 responses. Molecular Cell*,* 7 *(*5*),* 915–926. 10.1016/S1097-2765(01)00242-8; 11389839

[10] Topolska-Woś, A. M., Chazin, W. J., Filipek, A. (2016). CacyBP/SIP--Structure and variety of functions. Biochimica et Biophysica Acta*,* 1860 *(*1*),* 79–85. 10.1016/j.bbagen.2015.10.012; 26493724

[11] Bhattacharya, S., Lee, Y. T., Michowski, W., Jastrzebska, B., Filipek, A. et al. (2005). The modular structure of SIP facilitates its role in stabilizing multiprotein assemblies. Biochemistry*,* 44 *(*27*),* 9462–9471. 10.1021/bi0502689; 15996101

[12] Shi, H., Gao, Y., Tang, Y., Wu, Y., Gong, H. et al. (2014). CacyBP/SIP protein is important for the proliferation of human glioma cells. IUBMB Life*,* 66 *(*4*),* 286–291. 10.1002/iub.1263; 24740456

[13] Wang, N., Ma, Q., Wang, Y., Ma, G., Zhai, H. (2010). CacyBP/SIP expression is involved in the clinical progression of breast cancer. World Journal of Surgery*,* 34 *(*11*),* 2545–2552. 10.1007/s00268-010-0690-2; 20585948

[14] Ning, X., Sun, S., Hong, L., Liang, J., Liu, L. et al. (2007). Calcyclin-binding protein inhibits proliferation, tumorigenicity, and invasion of gastric cancer. Molecular Cancer Research*,* 5 *(*12*),* 1254–1262. 10.1158/1541-7786.MCR-06-0426; 18171983

[15] Rines, A. K., Burke, M. A., Fernandez, R. P., Volpert, O. V., Ardehali, H. (2012). Snf1-related kinase inhibits colon cancer cell proliferation through calcyclin-binding protein-dependent reduction of β-catenin. FASEB Journal*,* 26 *(*11*),* 4685–4695. 10.1096/fj.12-212282; 22874833 PMC3475258

[16] Chen, X., Han, G., Zhai, H., Zhang, F., Wang, J. et al. (2008). Expression and clinical significance of CacyBP/SIP in pancreatic cancer. Pancreatology*,* 8 *(*4–5*),* 470–477. 10.1159/000151774; 18765951

[17] Popovic, D., Vucic, D., Dikic, I. (2014). Ubiquitination in disease pathogenesis and treatment. Nature Medicine*,* 20 *(*11*),* 1242–1253. 10.1038/nm.3739; 25375928

[18] Niu, Y. L., Li, Y. J., Wang, J. B., Lu, Y. Y., Liu, Z. X. et al. (2016). CacyBP/SIP nuclear translocation regulates p27Kip1 stability in gastric cancer cells. World Journal of Gastroenterology*,* 22 *(*15*),* 3992–4001. 10.3748/wjg.v22.i15.3992; 27099442 PMC4823249

[19] Banales, J. M., Cardinale, V., Carpino, G., Marzioni, M., Andersen, J. B. et al. (2016). Expert consensus document: Cholangiocarcinoma: Current knowledge and future perspectives consensus statement from the European Network for the Study of Cholangiocarcinoma (ENS-CCA). Nature Reviews Gastroenterology & Hepatology*,* 13 *(*5*),* 261–280. 10.1038/nrgastro.2016.51; 27095655

[20] Munoz-Garrido, P., Rodrigues, P. M. (2019). The jigsaw of dual hepatocellular-intrahepatic cholangiocarcinoma tumours. Nature Reviews Gastroenterology & Hepatology*,* 16 *(*11*),* 653–655. 10.1038/s41575-019-0185-z; 31296968

[21] Lindnér, P., Rizell, M., Hafström, L. (2015). The impact of changed strategies for patients with cholangiocarcinoma in this millenium. HPB Surgery*,* 2015*,* 736049. 10.1155/2015/736049; 25788760 PMC4348584

[22] Spolverato, G., Kim, Y., Alexandrescu, S., Marques, H. P., Lamelas, J. et al. (2016). Management and outcomes of patients with recurrent intrahepatic cholangiocarcinoma following previous curative-intent surgical resection. Annals of Surgical Oncology*,* 23 *(*1*),* 235–243. 10.1245/s10434-015-4642-9; 26059651

[23] Lian, Y. F., Huang, Y. L., Zhang, Y. J., Chen, D. M., Wang, J. L. et al. (2019). CACYBP enhances cytoplasmic retention of P27(Kip1) to promote hepatocellular carcinoma progression in the absence of RNF41 mediated degradation. Theranostics*,* 9 *(*26*),* 8392–8408. 10.7150/thno.36838; 31754404 PMC6857042

[24] Forsburg, S. L. (2004). Eukaryotic MCM proteins: Beyond replication initiation. Microbiology and Molecular Biology Reviews*,* 68 *(*1*),* 109–131. 10.1128/mmbr.68.1.109-131.2004; 15007098 PMC362110

[25] Lei, M. (2005). The MCM complex: Its role in DNA replication and implications for cancer therapy. Current Cancer Drug Targets*,* 5 *(*5*),* 365–380. 10.2174/1568009054629654; 16101384

[26] Parker M. W., Botchan M. R., Berger J. M. (2017). Mechanisms and regulation of DNA replication initiation in eukaryotes. Critical Reviews in Biochemistry and Molecular Biology*,* 52 *(*2*),* 107–144. 10.1080/10409238.2016.1274717; 28094588 PMC5545932

[27] Romanowski P., Madine M. A. (1997). Mechanisms restricting DNA replication to once per cell cycle: The role of Cdc6p and ORC. Trends in Cell Biology*,* 7 *(*1*),* 9–10. 10.1016/S0962-8924(97)30077-4; 17708892

[28] Hennessy, K. M., Lee, A., Chen, E., Botstein, D. (1991). A group of interacting yeast DNA replication genes. Genes & Development*,* 5 *(*6*),* 958–969. 10.1101/gad.5.6.958; 2044962

[29] de Andrade, BA., León, J. E., Carlos, R., Delgado-Azañero, W., Mosqueda-Taylor, A. et al. (2013). Expression of minichromosome maintenance 2, Ki-67, and geminin in oral nevi and melanoma. Annals of Diagnostic Pathology*,* 17 *(*1*),* 32–36. 10.1016/j.anndiagpath.2012.05.001; 22652151

[30] Yousef, E. M., Furrer, D., Laperriere, D. L., Tahir, M. R., Mader, S. et al. (2017). MCM2: An alternative to Ki-67 for measuring breast cancer cell proliferation. Modern Pathology*,* 30 *(*5*),* 682–697. 10.1038/modpathol.2016.231; 28084344

[31] Czyzewska, J., Guzińska-Ustymowicz, K., Pryczynicz, A., Kemona, A., Bandurski, R. (2009). Immunohistochemical evaluation of Ki-67, PCNA and MCM2 proteins proliferation index (PI) in advanced gastric cancer. FFolia Histochemica et Cytobiologica*,* 47 *(*2*),* 289–296. 10.2478/v10042-009-0042-y; 19995716

[32] Yan, S., Li, A., Liu, Y. (2018). CacyBP/SIP inhibits the migration and invasion behaviors of glioblastoma cells through activating Siah1 mediated ubiquitination and degradation of cytoplasmic p27. Cell Biology International*,* 42 *(*2*),* 216–226. 10.1002/cbin.10889; 29024247

[33] Nusse, R., Clevers, H. (2017). Wnt/β-Catenin signaling, disease, and emerging therapeutic modalities. Cell*,* 169 *(*6*),* 985–999. 10.1016/j.cell.2017.05.016; 28575679

[34] Clevers, H., Nusse, R. (2012). Wnt/β-catenin signaling and disease. Cell*,* 149 *(*6*),* 1192–1205. 10.1016/j.cell.2012.05.012; 22682243

[35] Zhang, Y., Wang, X. (2020). Targeting the Wnt/β-catenin signaling pathway in cancer. Journal of Hematology & Oncology*,* 13 *(*1*),* 165. 10.1186/s13045-020-00990-3; 33276800 PMC7716495

[36] Zhang, G. F., Qiu, L., Yang, S. L., Wu, J. C., Liu, T. J. (2020). Wnt/β-catenin signaling as an emerging potential key pharmacological target in cholangiocarcinoma. Bioscience Reports*,* 40 *(*3*),* BSR20193353. 10.1042/BSR20193353; 32140709 PMC7953494

[37] Haonon, O., Rucksaken, R., Pinlaor, P., Pairojkul, C., Chamgramol, Y. et al. (2016). Upregulation of 14-3-3 eta in chronic liver fluke infection is a potential diagnostic marker of cholangiocarcinoma. Proteomics Clinical Applications*,* 10 *(*3*),* 248–256. 10.1002/prca.201500019; 26435198

[38] Haage, V., Semtner, M., Vidal, R. O., Hernandez, D. P., Pong, W. W. et al. (2019). Comprehensive gene expression meta-analysis identifies signature genes that distinguish microglia from peripheral monocytes/macrophages in health and glioma. Acta Neuropathologica Communications*,* 7 *(*1*),* 20. 10.1186/s40478-019-0665-y; 30764877 PMC6376799

